# Promising Candidate Prognostic Biomarkers in [^18^F]FDG PET Images: Evaluation in Independent Cohorts of Non–Small Cell Lung Cancer Patients

**DOI:** 10.2967/jnumed.123.266331

**Published:** 2024-04

**Authors:** Narinée Hovhannisyan-Baghdasarian, Marie Luporsi, Nicolas Captier, Christophe Nioche, Vesna Cuplov, Erwin Woff, Nadia Hegarat, Alain Livartowski, Nicolas Girard, Irène Buvat, Fanny Orlhac

**Affiliations:** 1 LITO U1288, Institut Curie, PSL University, Inserm, Orsay, France;; 2 Department of Nuclear Medicine, Institut Curie, Paris, France;; 3 Department of Nuclear Medicine, Institut Jules Bordet, Hôpital Universitaire de Bruxelles, Université Libre de Bruxelles, Brussels, Belgium;; 4 Institut du Thorax Curie-Montsouris, Institut Curie, Paris, France; and; 5 Paris Saclay Cancer Campus, UVSQ, Versailles, France

**Keywords:** [^18^F]FDG PET, lung cancer, oncology, immunotherapy, radiomics

## Abstract

The normalized distances from the hot spot of radiotracer uptake (SUV_max_) to the tumor centroid (NHOC) and to the tumor perimeter (NHOP) have recently been suggested as novel PET features reflecting tumor aggressiveness. These biomarkers characterizing the shift of SUV_max_ toward the lesion edge during tumor progression have been shown to be prognostic factors in breast and non–small cell lung cancer (NSCLC) patients. We assessed the impact of imaging parameters on NHOC and NHOP, their complementarity to conventional PET features, and their prognostic value for advanced-NSCLC patients. **Methods:** This retrospective study investigated baseline [^18^F]FDG PET scans: cohort 1 included 99 NSCLC patients with no treatment-related inclusion criteria (robustness study); cohort 2 included 244 NSCLC patients (survival analysis) treated with targeted therapy (93), immunotherapy (63), or immunochemotherapy (88). Although 98% of patients had metastases, radiomic features including SUVs were extracted from the primary tumor only. NHOCs and NHOPs were computed using 2 approaches: the normalized distance from the localization of SUV_max_ or SUV_peak_ to the tumor centroid or perimeter. Bland–Altman analyses were performed to investigate the impact of both spatial resolution (comparing PET images with and without gaussian postfiltering) and image sampling (comparing 2 voxel sizes) on feature values. The correlation of NHOCs and NHOPs with other features was studied using Spearman correlation coefficients (*r*). The ability of NHOCs and NHOPs to predict overall survival (OS) was estimated using the Kaplan–Meier method. **Results:** In cohort 1, NHOC and NHOP features were more robust to image filtering and to resampling than were SUVs. The correlations were weak between NHOCs and NHOPs (*r* ≤ 0.45) and between NHOCs or NHOPs and any other radiomic features (*r* ≤ 0.60). In cohort 2, the patients with short OS demonstrated higher NHOCs and lower NHOPs than those with long OS. NHOCs significantly distinguished 2 survival profiles in patients treated with immunotherapy (log-rank test, *P* < 0.01), whereas NHOPs stratified patients regarding OS in the targeted therapy (*P* = 0.02) and immunotherapy (*P* < 0.01) subcohorts. **Conclusion:** Our findings suggest that even in advanced NSCLC patients, NHOC and NHOP features pertaining to the primary tumor have prognostic potential. Moreover, these features appeared to be robust with respect to imaging protocol parameters and complementary to other radiomic features and are now available in LIFEx software to be independently tested by others.

## Visual Abstract

**Figure:**
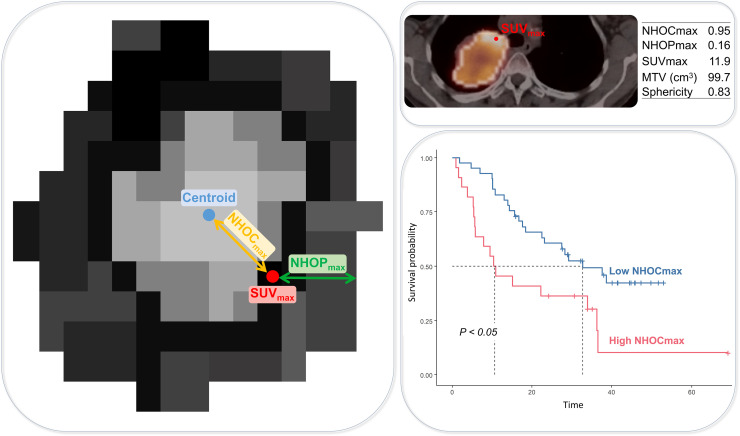

Among radiomic features derived from PET images, those reflecting the geometric characteristics of the metabolically active part of a tumor might have some prognostic value for survival. For instance, asymmetric and irregular tumor shapes on PET appear to be associated with high-grade neoplasms and thus with poor survival (1,2). Moreover, geometry-based features have been shown to be robust to imaging protocol parameters (3), which is an asset for translation in clinical settings. Recently, the normalized distance between the hot spot of radiotracer uptake (SUV_max_) and the tumor centroid (NHOC) has been introduced as a novel PET feature by Jiménez-Sánchez et al. (4), based on a mathematic model of solid-tumor growth. This model suggested that the maximum metabolic activity of a tumor (reflected by SUV_max_ in a PET image) is expected to increase and to move toward the lesion edge as the tumor grows. Another feature for evaluating the change in intratumor heterogeneity, called normalized SUV_max_ to perimeter distance (nSPD), has been proposed by Jiménez Londoño et al. (5), defined as the normalized closest distance between the maximum metabolic activity location (SUV_max_) and the tumor perimeter (NHOP). Both NHOC and nSPD were proposed as hallmarks of tumor aggressiveness. They were demonstrated to be associated with worse outcome in breast and advanced non–small cell lung cancer (NSCLC) patients, outperforming the conventional PET features such as SUVs (5). Originally, the nSPD was defined on the axial slice of the tumor that included the voxel with maximum activity (5). In our study, we computed NHOP considering the tumor in 3 dimensions to make it more comparable to NHOC.

The goal of the present study was thus to evaluate the robustness of NHOC and NHOP to image characteristics such as spatial resolution and voxel size, to determine their correlation with conventional PET features, and to evaluate their prognostic value in predicting survival in NSCLC patients.

## MATERIALS AND METHODS

### Patient Cohorts

The study was conducted in accordance with the Declaration of Helsinki and approved by the ethical board of Institut Curie, France (approval DATA200130) with a waiver of informed consent through the no-objection rule.

This retrospective study included 2 cohorts of a total of 343 patients with advanced, metastatic NSCLC treated at our institute between 2009 and 2021, who had undergone baseline [^18^F]FDG PET/CT before treatment initiation. The first cohort was used to analyze the robustness of NHOC and NHOP with respect to imaging parameters, whereas the second cohort was used to assess the performance of the features in predicting overall patient survival. Cohort 1 consisted of 99 patients, with no selection criteria regarding their subsequent treatment. Cohort 2 consisted of 244 patients and included 3 subcohorts of patients treated with either targeted therapy, namely tyrosine kinase inhibitors for epithelial growth factor receptor–mutated patients (93), or immunotherapy, namely pembrolizumab (alone or in combination with chemotherapy, 63 and 88, respectively). The inclusion criteria were as follows: baseline [^18^F]FDG PET/CT scan available in the PACS, detectability of primary lesion on the PET scan, and at least 1-y follow-up of the patient. For all patients, survival data were retrieved. Patient characteristics such as age, sex, histologic subtype, and stage are summarized in Table 1.

**TABLE 1.** Patient Characteristics

Characteristic	Cohort 1	Cohort 2
Number of patients		
Total	99	244
Male	55 (56%)	122 (50%)
Female	44 (44%)	122 (50%)
Age (y)		
Mean ± SD	66.3 ± 10.2	65.4 ± 10.1
Range	35–86	35–87
NSCLC subtype		
Adenocarcinoma	58 (58%)	196 (80%)
Squamous cell carcinoma	28 (28%)	24 (10%)
Other	13 (13%)	24 (10%)
Stage		
III	50 (51%)	5 (2%)
VI	49 (49%)	239 (98%)

Data are number with percentage in parentheses, except for age.

### Image Database and Image Processing

The [^18^F]FDG PET/CT scans were acquired at different centers using 13 scanners from different vendors (Supplemental Table 1; supplemental materials are available at http://jnm.snmjournals.org). The volume of interest (VOI) encompassing the primary tumor was defined on the axial views of the PET scan by an experienced nuclear medicine physicist (8 y of experience) under the supervision of a nuclear medicine physician (10 y of experience). The metabolically active volume of the lesion was delineated automatically using a threshold set to 40% of SUV_max_. A morphologic closing operation was applied to include internal necrotic areas in the VOI if necessary. To investigate the impact of tumor delineation on NHOC and NHOP and radiomic feature values, 30 scans randomly chosen from cohort 1 were segmented independently by a second observer (with 10 y of experience). All images were resampled to a fixed 4 × 4 × 4 mm voxel size, and the intensity was discretized with a fixed bin width of 0.31 SUV and 192 gray levels between 0 and 60 SUV (6). Thirteen features were then extracted from the VOI, including 6 conventional PET features (SUV_max_, SUV_peak_ [SUV within a 1 cm^3^ sphere with maximum average uptake (7)], SUV_mean_, SUV_min_, metabolic tumor volume [MTV], and total lesion glycolysis), along with a shape feature (sphericity) and 6 textural features (joint entropy log10, inverse difference moment, short run emphasis, long run emphasis, low gray-level zone emphasis, and high gray-level zone emphasis) (definitions available at https://www.lifexsoft.org/index.php/resources/documentation). These indices were previously identified (8) as not strongly correlated features and were found to be the most robust with respect to tumor segmentation method. In addition, NHOCs and NHOPs were computed using 2 approaches: the distance from the localization of SUV_max_ or SUV_peak_ to the tumor centroid (and perimeter, respectively) divided by the radius of a hypothetical sphere having the same volume as the tumor, thus yielding dimensionless quantities: maximum NHOC (NHOC_max_), peak NHOC (NHOC_peak_), maximum NHOP (NHOP_max_), and peak NHOP (NHOP_peak_) (Supplemental Fig. 1).

For the robustness study using cohort 1, 2 other sets of PET images were generated to evaluate the effects of spatial resolution and voxel size on image-derived features. The first set was obtained by postfiltering (gaussian, SD [σ] of 2, 3, and 4 mm; corresponding to a full width at half maximum of approximately 5, 7, and 9 mm, respectively) the original images of cohort 1. The second set was obtained by resampling the original images of cohort 1 to a 2 × 2 × 2 mm fixed voxel size. Cohort 2 was used for survival analyses. If the VOI was too small (diameter < 12 mm), SUV_peak_ and therefore NHOC_peak_ and NHOP_peak_ could not be calculated and were replaced by SUV_max_ and by NHOC_max_ and NHOP_max_, respectively. Additionally, to investigate the impact of necrotic foci on NHOCs and NHOPs and other radiomic feature values, all 244 lesions of cohort 2 were labeled as necrotic or nonnecrotic using visual assessment of PET images by an experienced nuclear medicine physicist under the supervision of a nuclear medicine physician.

Image processing, tumor segmentation, and feature extraction were performed with LIFEx (version 7.4.0, www.lifexsoft.org) (9), which is an Image Biomarker Standardisation Initiative–compliant software (10).

### Statistical Analysis

Statistical analyses were conducted using R software (R Core Team 2021).

For the robustness study, before-and-after comparison analyses were performed using the Bland–Altman method with 95% limits of agreement (±1.96 SDs) to investigate the impact of spatial resolution (with vs. without postfiltering) and voxel size (2 × 2 × 2 mm vs. 4 × 4 × 4 mm) on NHOCs, NHOPs, and SUVs.

To characterize the correlations between features, the Spearman rank correlation coefficient (*r*) was computed between each pair of features.

The impact of tumor delineation on radiomic feature values was assessed using the 30 lesions segmented by 2 observers, by calculating the intraclass correlation coefficient based on the 1-way model with agreement-type and single-score settings.

The ability of NHOCs, NHOPs, and other radiomic features to predict overall survival (OS) was investigated using Kaplan–Meier analysis, where the duration of follow-up was defined as the time between the pretreatment PET scan and the date of death or last day of follow-up. The significance of differences between survival curves was assessed by log-rank testing, with a *P* value of less than 0.05 defined as statistically significant. The stratification involved the maximally selected rank statistics methodology, which assesses 2-sample rank statistics of all possible cutoffs and selects the optimal one. The Harrell C-index was also used to evaluate the ability of each feature to predict survival. The relevant predictors after Kaplan–Meier analysis were further combined in pairs for multivariable analysis, where 3 risk categories were defined on the basis of whether the feature’s high or low value was associated with good or poor outcome (e.g., high MTV associated with worse survival). The pairwise comparisons between survivals of the risk groups were calculated using log-rank tests, including the Benjamini–Hochberg method for *P* value adjustment.

The impact of the presence of visible necrosis on PET images on NHOCs and NHOPs and other imaging features was evaluated by Wilcoxon rank testing by comparing feature values between tumors with and without necrotic cores. Moreover, we compared the survival prediction accuracy based on log-rank testing with and without the necrosis included in the tumor region, that is, no morphologic closing operation applied after thresholding.

## RESULTS

The statistical distribution of the NHOCs and NHOPs is shown in Table 2 and compared with those of the other 13 features. According to MTV, the tumor size ranged from 0.3 to 1,019.6 cm^3^. The mean values were 0.62 (range, 0.09–1.83) for NHOC_max_, 0.54 (range, 0.04–1.83) for NHOC_peak_, 0.26 (range, 0.05–1.26) for NHOP_max_, and 0.27 (range, 0.05–0.63) for NHOP_peak_.

**TABLE 2.** Statistical Distribution of Features for Cohort 2 (*n* = 244 Patients)

Variable	Mean	SD	Range
SUV_min_	3.8	2.6	0.0–17.0
SUV_max_	13.3	7.2	1.8–51.8
SUV_peak_	11.4	6.1	2.6–40.0
SUV_mean_	7.5	4.1	1.2–30.9
NHOC_max_	0.615	0.290	0.087–1.833
NHOC_peak_	0.538	0.292	0.042–1.833
NHOP_max_	0.255	0.155	0.049–1.264
NHOP_peak_	0.271	0.131	0.053–0.628
MTV (cm3)	57.1	112.0	0.3–1019.6
Total lesion glycolysis	396.9	810.2	0.9–8557.9
Sphericity	0.763	0.110	0.320–0.903
Joint entropy log10	3.586	0.332	2.874–4.189
Inverse difference moment	0.040	0.024	0.014–0.227
Short run emphasis	0.992	0.007	0.935–1.000
Long run emphasis	1.034	0.041	1.000–1.462
Low gray-level zone emphasis	0.010	0.010	0.000–0.049
High gray-level zone emphasis	7,374.3	2,228.4	1,338.7–13,788.2

### Robustness Study

**FIGURE 1.**
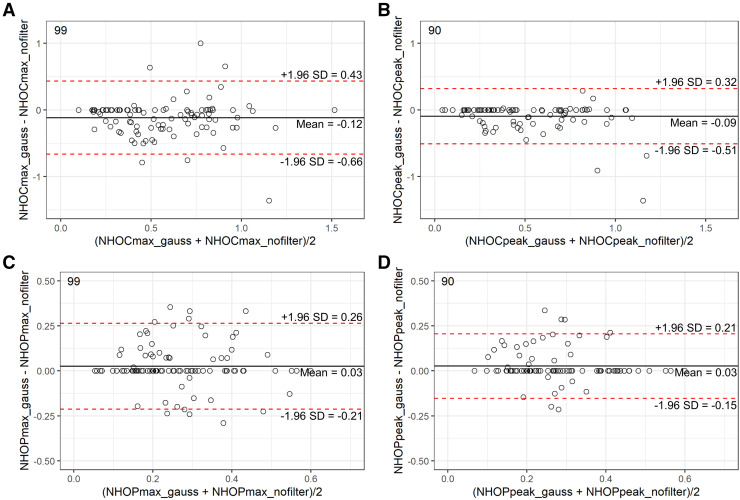
Bland–Altman plots for cohort 1, showing concordance between feature values extracted from PET images (4-mm voxel size) before (suffix _nofilter on the graphs) and after gaussian postfiltering (σ = 4 mm, suffix _gauss) for NHOC_max_ (A), NHOC_peak_ (B), NHOP_max_ (C), and NHOP_peak_ (D). Limits of agreement (95%) are shown as dotted red lines, and bias is shown as solid black line. Numbers in top-left corner of each graph correspond to number of measurements available.

In cohort 1, Bland–Altman analysis showed that NHOCs were little affected by image postfiltering (mean difference of −0.12 for NHOC_max_ and −0.09 for NHOC_peak_, with the greatest smoothing with a σ of 4 mm [Fig. 1]; similar results with smaller filter sizes [Supplemental Figs. 2 and 3]) and even less by the voxel size (mean difference of 0.02 and 0.003, respectively [Supplemental Fig. 4]). The mean difference was only 0.03 for both NHOP_max_ and NHOP_peak_ when the postfiltering was applied (σ of 4 mm) and was −0.02 and −0.07, respectively, when the voxel size was varied. For both features, the measurements based on SUV_max_ demonstrated greater variability (larger CI) than those based on SUV_peak_. For comparison purposes, SUV_peak_ and SUV_max_ were more affected by postfiltering and voxel size than the NHOC and NHOP features, with increasing systematic differences observed for greater SUVs (Supplemental Figs. 2–5).

Comparing feature values obtained for VOIs drawn by 2 observers, the reproducibility was excellent (intraclass correlation coefficient ≥ 0.8) for NHOCs and NHOPs and for most features (Supplemental Table 2).

**FIGURE 2.**
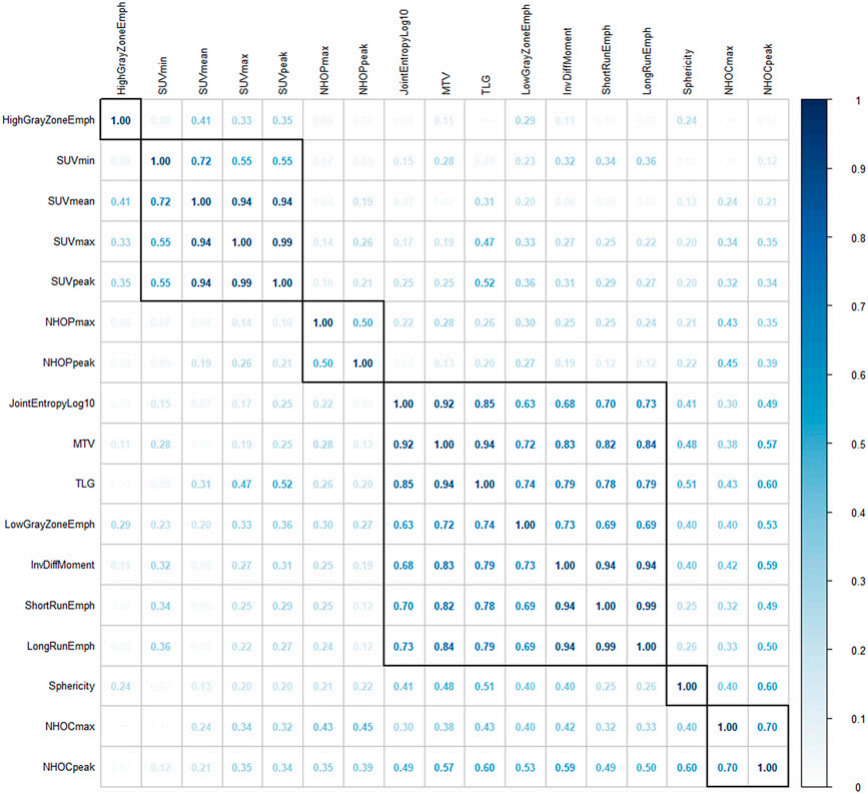
Correlogram from cohort 1, showing absolute Spearman correlation coefficient between each pair of radiomic features. Rectangles are generated on graph according to hierarchic clustering. HighGrayZoneEmph = high gray-level zone emphasis; InvDiffMoment = inverse difference moment; JointEntropyLog10 = joint entropy log10; LongRunEmph = long run emphasis; LowGrayZoneEmph = low gray-level zone emphasis; ShortRunEmph = short run emphasis; TLG = total lesion glycolysis.

As illustrated in the correlogram (Fig. 2), NHOCs and NHOPs did not correlate strongly with any of the other features, with correlations always less than 0.60 and 0.30 in absolute value for NHOCs and NHOPs, respectively. The correlations between NHOCs and NHOPs did not exceed 0.45.

### Survival Analysis

Among the 244 patients included in cohort 2, SUV_peak_ and therefore NHOC_peak_ and NHOP_peak_ could be calculated in 218 (89%) and were replaced by SUV_max_ and by NHOC_max_ and NHOP_max_, respectively, in the remaining 26 patients, who had a primary tumor less than 12 mm in largest diameter.

**FIGURE 3.**
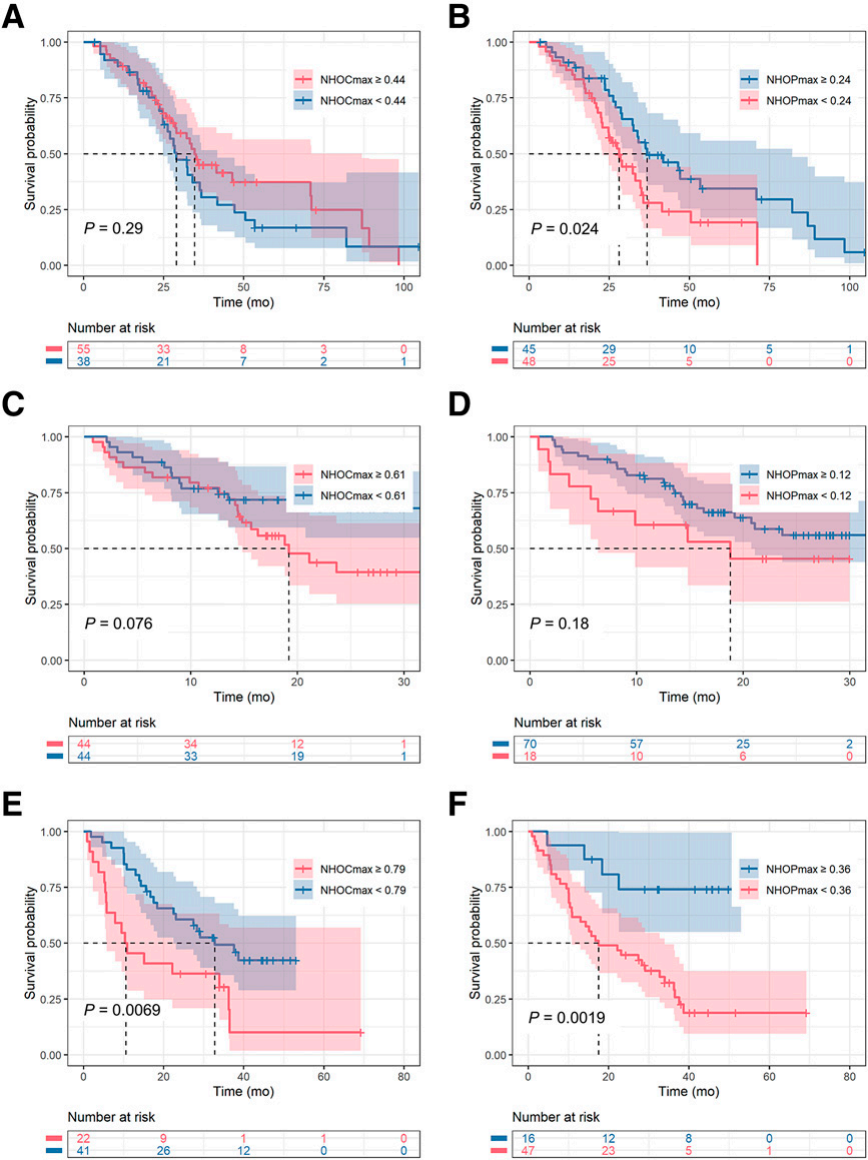
Kaplan–Meier OS curves with best cutoffs for NHOC_max_ and NHOP_max_ for patients treated by targeted therapy (A and B), immunochemotherapy (C and D), and immunotherapy only (E and F), based on baseline [^18^F]FDG PET scans.

NHOCs identified 2 survival profiles in patients treated with immunotherapy only (*P* value of log-rank test < 0.01; cutoffs, 0.79 for NHOC_max_ and 0.50 for NHOC_peak_ [Fig. 3; Table 3]). In the other 2 subcohorts, the distinction of patients with low and high OS did not reach significance. NHOPs significantly distinguished long- from short-OS patients in the targeted therapy and immunotherapy groups (Table 3). The highest C-index for NHOCs and NHOPs was 0.58 for NHOC_max_, 0.61 for NHOC_peak_, 0.57 for NHOP_max_, and 0.59 for NHOP_peak_ (Table 3). For other features, the greatest observed C-indexes were for MTV (0.62) and for sphericity (0.69), both in the immunotherapy group. Supplemental Figure 6 shows the results for NHOC_max_, NHOP_max_, MTV, and sphericity, where for each feature the survival curves of the 3 treatment groups are overlaid on a single graph using the same cutoff (0.80 for NHOC_max_, 0.37 for NHOP_max_, 30.4 cm^3^ for MTV, and 0.73 for sphericity). For each feature, the common cut point was determined according to the optimal separation between patients concerning long and short OS when considering all patients of cohort 2 regardless of their treatment. In multivariable analysis, none of the feature combinations significantly distinguished the survival distributions of the patients according to 3 risk categories (Supplemental Table 3). Nevertheless, better intercategory distinction was observed in the immunotherapy group with the combination of NHOC_max_/NHOP_max_ and sphericity, for instance (Supplemental Fig. 7).

**TABLE 3.** Survival Analyses with Kaplan–Meier Estimate of OS and C-Index

	Targeted therapy				Immunochemotherapy				Immunotherapy			
Feature	Cutoff	*P*	Short-OS	C-index	Cutoff	*P*	Short-OS	C-index	Cutoff	*P*	Short-OS	C-index
SUV_min_	1.4	0.030*	−	0.499	5.2	0.031*	−	0.468	5.4	0.004*	+	0.488
SUV_max_	10.5	0.007*	+	0.577	9.1	0.230		0.460	18.2	0.110		0.517
SUV_peak_	9.1	0.023*	+	0.571	12.9	0.230		0.462	11.9	0.190		0.520
SUV_mean_	6.0	0.019*	+	0.563	8.4	0.068		0.471	12.4	0.004*	+	0.472
NHOC_max_	0.435	0.290		0.477	0.608	0.076		0.540	0.793	0.007*	+	0.581
NHOC_peak_	0.521	0.220		0.496	0.475	0.150		0.500	0.502	0.005*	+	0.614
NHOP_max_	0.238	0.024*	−	0.572	0.115	0.180		0.510	0.361	0.002*	−	0.541
NHOP_peak_	0.238	0.008*	−	0.540	0.192	0.043*	+	0.449	0.468	0.013*	−	0.586
MTV (cm3)	55.7	0.029*	+	0.529	21.4	0.006*	+	0.592	21.0	0.003*	+	0.624
Total lesion glycolysis	84.5	0.170		0.542	53.9	0.014*	+	0.577	193.6	0.002*	+	0.616
Sphericity	0.784	0.095		0.502	0.721	0.017*	−	0.546	0.830	<0.001*	−	0.690
Joint entropy log10	3.725	0.140		0.532	3.695	0.007*	+	0.607	3.665	0.068		0.538
Inverse difference moment	0.04	0.030*	+	0.488	0.036	0.022*	+	0.455	0.039	0.025*	+	0.421
Short run emphasis	0.99	0.071		0.480	0.993	0.015*	−	0.449	0.991	0.024*	−	0.433
Long run emphasis	1.04	0.035*	+	0.486	1.026	0.015*	+	0.459	1.034	0.024*	+	0.431
Low gray-level zone emphasis	0.003	0.011*	−	0.563	0.004	0.150		0.529	0.004	<0.001*	−	0.594
High gray-level zone emphasis	6183	0.042*	+	0.570	5776	0.034*	−	0.433	6695	0.013*	−	0.406

* *P* < 0.05 (log-rank test).

+ or − = higher or lower than cutoff feature value associated with poor survival.

**FIGURE 4.**
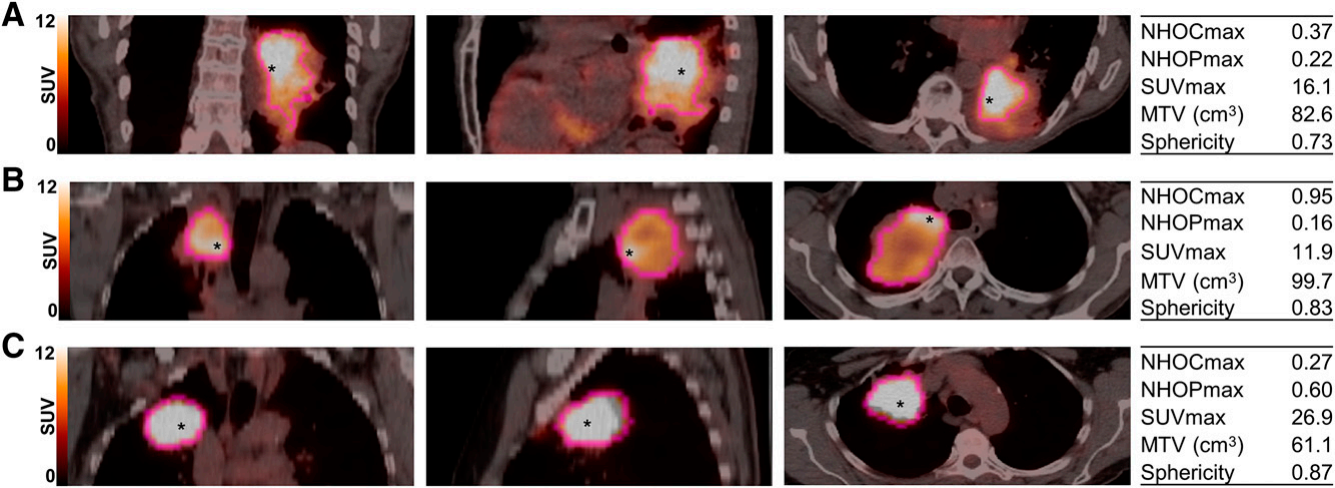
Examples of tumor and corresponding feature values on [^18^F]FDG PET/CT coronal, sagittal, and axial slices from patients with different survivals: 17.6 mo (A), 35.1 mo (B), and alive at 49.8 mo (C). Position of hottest voxel is indicated with asterisk.

As reported in Supplemental Table 4, the lesions labeled as necrotic (68/244, 28%) or nonnecrotic based on visual evaluation of PET images yielded significantly different values (Wilcoxon test) for all features except SUV_mean_, sphericity, inverse difference moment, short run emphasis, long run emphasis, low gray-level zone emphasis, and high gray-level zone emphasis. The lesions with a necrotic core exhibited higher NHOCs and lower NHOPs than those without necrosis (Supplemental Fig. 8). This is illustrated in Figure 4, which shows representative PET/CT scans of tumors with different NHOCs and NHOPs. However, the accuracy of survival predictions with NHOCs and NHOPs was little affected by exclusion of the necrotic areas in the tumor VOIs (Supplemental Fig. 9; Fig. 3; Supplemental Table 5).

## DISCUSSION

Several imaging features, such as SUV and MTV, are widely studied for the management of cancer patients (11,12). More sophisticated radiomic features are also extensively investigated and considered for inclusion in classification, prognostic, and predictive models. However, none of the advanced imaging features has yet gained wide acceptance, partly because of their challenging intuitive interpretation and lack of easy-to-understand reference values. NHOC and nSPD are recently introduced model-informed PET metrics, characterizing the drift of highly proliferative cells toward the tumor periphery (4,5), which can thus be related to tumor growth, and are easy to calculate and to interpret. They were defined as the distance from the localization of the [^18^F]FDG hot spot to the tumor centroid or perimeter. To avoid the size dependence of these metrics, they were divided by the radius of a hypothetical sphere having the same volume as the tumor. Although their prognostic value has been demonstrated in breast cancer and NSCLC (4,5), to the best of our knowledge, they have not been confirmed yet in any independent study. Therefore, the current study first investigated the sensitivity of NHOC and NHOP (the 3-dimensional [3D] version of the nSPD feature) to image spatial resolution, spatial sampling, and tumor delineation and then studied their prognostic value in independent cohorts of NSCLC patients.

For each biomarker, 2 measurement approaches were used: NHOC_max_, as initially modeled and reported by Jiménez-Sánchez et al. (4), and NHOC_peak_, as introduced by Jiménez Londoño et al. (5). NHOP_max_ is a 3D surrogate of the already reported feature (5), and NHOP_peak_ is an alternative of the SUV_max_-based NHOP measurement. Because NHOC_peak_ and NHOP_peak_ calculations require the tumor to be larger than 1 cm^3^ so that SUV_peak_ can be calculated, they were replaced by NHOC_max_ and NHOP_max_ for tumors less than 12 mm in diameter.

Our results showed that NHOCs and NHOPs varied less with spatial resolution and image sampling than did SUV_max_ and SUV_peak_. Given that both SUV_max_ and SUV_peak_ are widely used although they are known to depend on image properties, our results suggest that NHOCs and NHOPs are usable even in multicenter studies, where the image quality often varies, and will not be strongly affected by imaging conditions. Another limiting issue with novel radiomic features is that they often correlate closely with existing ones, hence not bringing clear complementary information. We thus checked whether NHOCs and NHOPs correlated with existing features, in particular with shape features, as they can be seen as geometric features (Supplemental Fig. 1). The correlation analysis demonstrated that NHOCs and NHOPs correlated strongly with neither sphericity (*r* ≤ 0.60) nor with conventional PET features such as SUV (*r* ≤ 0.35) and MTV (*r* ≤ 0.57) or textural features (*r* ≤ 0.59), confirming their potential added value compared with widely investigated radiomic features. The correlations between NHOCs and NHOPs were also weak (Fig. 2), as these variables would be perfectly negatively correlated only if the tumors were perfectly spheric. Last, for a radiomic feature to be potentially useful, its values should not heavily depend on accurate tumor delineation. We therefore compared the feature values obtained when 2 different observers drew the VOI and observed an intraclass correlation coefficient greater than 0.8 for NHOCs and NHOPs, confirming these as suitable PET biomarker candidates. However, the observers used the same semiautomatic isocontouring (40%) approach on the manually delineated VOIs, and intraclass correlation coefficients might have been smaller had the segmentation methods been different.

The orders of magnitude of NHOCs found in our study are quite similar to the ones reported in the literature (NHOC_max_, 0.62 ± 0.29 in our study vs. 0.43 ± 0.20 in the literature (4); NHOC_peak_, 0.54 ± 0.29 in our study vs. 0.34 ± 0.20 in the literature (5)), facilitating their interpretation. No direct comparison could be made between reported nSPDs and our NHOPs because of differences in the definition of distance to perimeter (2-dimensional vs. 3D). For comparison purposes, the maximum nSPDs were similar in the present and previous (5) research, with mean values of 0.26 ± 0.16 and 0.40 ± 0.12, respectively. However, for tumor characterization we recommend the 3D approach (NHOP) to be consistent with the NHOC feature.

The differences in the cohorts in the previous (4,5) and present studies are major, preventing meaningful comparisons of feature values. Indeed, the 2 published studies included only surgically treated patients with different adjuvant treatment regimens, whereas in our study the patients of cohort 2 were not operable and were treated using various approaches. Moreover, the dataset of Jiménez-Sánchez et al. (4) was based on inclusion criteria (e.g., a tumor size of at least 2 cm, absence of distant metastases, and a tumor SUV twice higher than background SUV) different from ours (detectability of the primary tumor on the PET images).

In survival analysis, the patients with higher NHOCs had shorter OS than those with lower NHOCs. Inversely, patients with lower NHOPs were associated with worse survival (except for NHOP_peak_ in the immunochemotherapy subcohort, for which the *P* value was 0.043 [Table 3]). These results are consistent with those of the previous studies. Jiménez-Sánchez et al. (4) reported an NHOC_max_ of 0.64 as the best cutoff to identify 2 survival profiles, which led to a C-index of 0.75 for OS. Here, a very similar cutoff (0.61) was obtained in the immunochemotherapy group, which, however, did not reach statistical significance in distinguishing patient survival, with the C-index result substantially lower in the present study (0.54 vs. 0.75). A higher cutoff of NHOC_max_ (0.79) stratified patients regarding OS in the immunotherapy group. Jiménez Londoño et al. (5) investigated NHOC_peak_, instead of NHOC_max_, with higher values of NHOC_peak_ (0.39 ± 0.21) being significantly associated with short-term mortality, defined as OS of less than 36 mo. In our study, an NHOC_peak_ of 0.50 significantly distinguished long- from short-OS patients in the immunotherapy group with a median follow-up of 24.2 mo. Jiménez Londoño et al. have also investigated nSPD as an alternative approach for measuring the displacement of the [^18^F]FDG hot spot (SUV_max_) from the center of the tumor to its periphery. This biomarker was reported as a significant prognostic factor for OS (a lower nSPD was significantly associated with short-term mortality) and correlated strongly with tumor histologic type and TNM stage. In the present study, we introduced the 3D surrogate of this biomarker, NHOP_max_, and its other variant, NHOP_peak_. Optimized cutoffs enabled patient stratification as a function of their OS with NHOP_max_ and NHOP_peak_ in the targeted therapy and immunotherapy groups. As a supplement, we considered C-index and OS estimates with the nSPD approach. Even if its prediction performances were similar with NHOP in all therapy groups (C-index, 0.51–0.57 for NHOP_max_ and 0.53–0.58 for maximum nSPD [Supplemental Fig. 10; Supplemental Table 6]), we recommend the 3D approach, which is in line with the other features such as MTV or NHOC calculated in 3 dimensions. According to our results, NHOPs outperformed NHOCs in predicting survival in 2 treatment groups, whereas NHOCs predicted survival only in the immunotherapy group. NHOC_max_ and NHOP_max_ were retained for further multivariate analysis in combination with the basic features such as SUV_max_, MTV, and sphericity. The best intercategory distinction was observed in the immunotherapy group with NHOC_max_–sphericity and NHOP_max_–sphericity pairs, which, however, could not significantly distinguish the survival distributions of the patients according to 3 risk categories.

Tumors with a necrotic core demonstrated significantly higher NHOCs and lower NHOPs. However, the survival outcomes were little affected by exclusion of the necrotic areas in the tumor VOIs. This is consistent with results published by Noortman et al. (13) for other radiomic features.

To our knowledge, this work was the first to compare the robustness and prognostic ability of NHOC_max_, NHOP_max_, NHOC_peak_, and NHOP_peak_. These features appeared equivalent with their maximum and peak approaches; however, NHOC_peak_ and NHOP_peak_ might be preferable to NHOC_max_ and NHOP_max_ for greater robustness to noise in PET images.

Similar to SUV, NHOCs and NHOPs pertain to a single lesion. When multiple lesions are present, the best way to account for NHOCs and NHOPs in all lesions remains to be investigated.

Our study had limitations. We investigated the prognostic value of NHOCs and NHOPs calculated in the primary tumor only, whereas 98% of patients had metastases. However, the results suggest that even in advanced-stage NSCLC patients, NHOC and NHOP features pertaining to the primary tumor have some prognostic value. Combining the NHOCs and NHOPs of the primary tumor with patient-level features such as total whole-body MTV and maximal distance between tumor foci—2 widely investigated whole-body radiomic features (14,15)—is under way. The minimal tumoral volume needed for meaningful measurement of these features should also be investigated to avoid the partial-volume effect related to spatial resolution and respiratory motion.

Finally, further studies are needed to determine the added value of NHOCs and NHOPs for different types of cancer and clinical questions of interest, as well as for tracers different from [^18^F]FDG. The dynamic changes in NHOCs and NHOPs could also be worth studying, as such changes may have the potential to predict treatment response. To encourage the PET community to reproduce our findings and to investigate NHOCs and NHOPs under various conditions, both biomarkers are now offered as additional radiomic features in the free LIFEx software (9).

## CONCLUSION

We validated the prognostic value of NHOC extracted from baseline [^18^F]FDG PET images of patients with NSCLC, independently of the original work of Jiménez-Sánchez et al. (4). We introduced the 3D surrogate of nSPD originally proposed by Jiménez Londoño et al. (5). Our findings confirmed the prognostic potential of NHOC and NHOP features when pertaining to the primary tumor in NSCLC patients. The prognostic significance of NHOC was obvious in the immunotherapy-treated patients (high NHOC associated with short OS), whereas low NHOP was associated with poor survival in the targeted therapy and immunotherapy groups. We demonstrated the robustness of NHOCs and NHOPs to postfiltering and voxel size resampling and their complementarity to SUVs, MTV, total lesion glycolysis, sphericity, and other commonly reported texture features. We encourage the PET community to include NHOCs and NHOPs in their PET image analysis to further evaluate their relevance for tumor characterization.

## DISCLOSURE

This work was supported by Fondation ARC (TIPIT project SIGNIT202001322) and by Fondation Janssen Horizon (PANACEE project). No other potential conflict of interest relevant to this article was reported.

## Notes

KEY POINTS **QUESTION:** Are NHOC and NHOP robust and prognostic PET biomarkers for lung cancer patients? **PERTINENT FINDINGS:** NHOC and NHOP biomarkers are robust to postfiltering of PET images and voxel size. They do not substantially correlate with other radiomic features. Depending on the treatment, they distinguished long- from short-OS lung cancer patients on baseline [^18^F]FDG PET images. **IMPLICATION FOR PATIENT CARE:** NHOC and NHOP are robust and prognostic biomarkers that deserve further evaluation in radiomic studies.

## References

[1] Kirienko M Gallivanone F Sollini M. FDG PET/CT as theranostic imaging in diagnosis of non-small cell lung cancer. Front Biosci (Landmark Ed). 2017;22:1713–1723. 28410141 10.2741/4567

[2] Apostolova I Ego K Steffen IG. The asphericity of the metabolic tumour volume in NSCLC: correlation with histopathology and molecular markers. Eur J Nucl Med Mol Imaging. 2016;43:2360–2373. 27470327 10.1007/s00259-016-3452-z

[3] Shiri I Rahmim A Ghaffarian P Geramifar P Abdollahi H Bitarafan-Rajabi A. The impact of image reconstruction settings on ^18^F-FDG PET radiomic features: multi-scanner phantom and patient studies. Eur Radiol. 2017;27:4498–4509. 28567548 10.1007/s00330-017-4859-z

[4] Jiménez-Sánchez J Bosque JJ Jiménez Londoño GA. Evolutionary dynamics at the tumor edge reveal metabolic imaging biomarkers. Proc Natl Acad Sci USA. 2021;118:e2018110118. 33536339 10.1073/pnas.2018110118 PMC8017959

[5] Jiménez Londoño GA Vicente AMG Bosque JJ. SUVmax to tumor perimeter distance: a robust radiomics prognostic biomarker in resectable non-small cell lung cancer patients. Eur Radiol. 2022;32:3889–3902. 35133484 10.1007/s00330-021-08523-3

[6] Orlhac F Nioche C Klyuzhin I Rahmim A Buvat I. Radiomics in PET imaging: a practical guide for newcomers. PET Clin. 2021;16:597–612. 34537132 10.1016/j.cpet.2021.06.007

[7] Wahl RL Jacene H Kasamon Y Lodge MA. From RECIST to PERCIST: evolving considerations for PET response criteria in solid tumors. J Nucl Med. 2009;50(suppl 1):122S–150S. 19403881 10.2967/jnumed.108.057307 PMC2755245

[8] Orlhac F Soussan M Maisonobe JA Garcia CA Vanderlinden B Buvat I. Tumor texture analysis in ^18^F-FDG PET: relationships between texture parameters, histogram indices, standardized uptake values, metabolic volumes, and total lesion glycolysis. J Nucl Med. 2014;55:414–422. 24549286 10.2967/jnumed.113.129858

[9] Nioche C Orlhac F Boughdad S. LIFEx: a freeware for radiomic feature calculation in multimodality imaging to accelerate advances in the characterization of tumor heterogeneity. Cancer Res. 2018;78:4786–4789. 29959149 10.1158/0008-5472.CAN-18-0125

[10] Zwanenburg A Vallières M Abdalah MA. The Image Biomarker Standardization Initiative: standardized quantitative radiomics for high-throughput image-based phenotyping. Radiology. 2020;295:328–338. 32154773 10.1148/radiol.2020191145 PMC7193906

[11] Sharma A Mohan A Bhalla A. Role of metabolic tumor volume (MTV) and standardized uptake value (SUV) based parameters derived from whole body (WB) ^18^F-FDG PET/CT in interim treatment response assessment of NSCLC [abstract]. J Nucl Med. 2019;60(suppl 1):1326.

[12] Ulaner GA Eaton A Morris PG. Prognostic value of quantitative fluorodeoxyglucose measurements in newly diagnosed metastatic breast cancer. Cancer Med. 2013;2:725–733. 24403238 10.1002/cam4.119 PMC3892804

[13] Noortman WA Vriens D Mooij CDY. The influence of the exclusion of central necrosis on [^18^F]FDG PET radiomic analysis. Diagnostics (Basel). 2021;11:1296. 34359379 10.3390/diagnostics11071296 PMC8304274

[14] Dall’Olio FG Calabrò D Conci N. Baseline total metabolic tumour volume on 2-deoxy-2-[^18^F]fluoro-d-glucose positron emission tomography-computed tomography as a promising biomarker in patients with advanced non-small cell lung cancer treated with first-line pembrolizumab. Eur J Cancer. 2021;150:99–107. 33892411 10.1016/j.ejca.2021.03.020

[15] Girum KB Rebaud L Cottereau AS. ^18^F-FDG PET maximum-intensity projections and artificial intelligence: a win-win combination to easily measure prognostic biomarkers in DLBCL patients. J Nucl Med. 2022;63:1925–1932. 35710733 10.2967/jnumed.121.263501 PMC9730929

